# “COVID knocked me straight into the dirt”: perspectives from people experiencing homelessness on the impacts of the COVID-19 pandemic

**DOI:** 10.1186/s12889-022-13748-y

**Published:** 2022-07-12

**Authors:** Natalia M. Rodriguez, Rebecca G. Martinez, Rebecca Ziolkowski, Cealia Tolliver, Hope Young, Yumary Ruiz

**Affiliations:** 1 Department of Public Health, College of Health and Human Sciences, Purdue University, West Lafayette, IN USA; 2 Regenstrief Center for Healthcare Engineering, Purdue University, West Lafayette, IN USA; 3 Department of Anthropology, College of Liberal Arts, Purdue University, West Lafayette, Indiana USA; 4 Department of Pharmacy Practice, College of Pharmacy, Purdue University, West Lafayette, Indiana USA

**Keywords:** COVID-19, Homelessness, Health disparities, Community-based participatory research, Socio-ecological model, Disaster response, Pandemic response

## Abstract

**Background:**

People experiencing homelessness are uniquely susceptible and disproportionately affected by the impacts of the COVID-19 pandemic. Understanding context-specific challenges, responses, and perspectives of people experiencing homelessness is essential to improving pandemic response and mitigating the long-term consequences of the pandemic on this vulnerable population.

**Methods:**

As part of an ongoing community-based participatory research study in partnership with a homeless service organization in Indiana, semi-structured interviews were conducted with a total of 34 individuals experiencing homelessness between January and July 2021. Guided by the NIMHD Health Disparities Research Framework, which builds on the socio-ecological model, data was thematically coded using Nvivo12 qualitative coding software and themes were organized by levels of influence (individual, interpersonal, community, societal) and domains of influence (biological, behavioral, physical/built environment, sociocultural environment, health care system).

**Results:**

Narratives revealed numerous and compounding factors affecting COVID-19 risks and health outcomes among people experiencing homelessness across all levels and domains of influence. At the individual level, people experiencing homelessness face unique challenges that heightened their susceptibility to COVID-19, including pre-existing physical and mental health conditions, substance use and behavioral health risks, socioeconomic precarity, and low health literacy and COVID-related knowledge. At the interpersonal level, poor communication between people experiencing homelessness and service providers led to limited understanding of and poor compliance with COVID safety measures. At the community level, closures and service disruptions restricted access to usual spaces and resources to meet basic needs. At a policy level, people experiencing homelessness were disregarded in ways that made pandemic relief resources largely inaccessible to them.

**Conclusions:**

Our findings reveal important and mitigable issues with ongoing pandemic response efforts in homeless populations through direct, first-hand accounts of their experiences during COVID-19. These insights offer opportunities for multilevel interventions to improve outreach, communication, and impact mitigation strategies for people experiencing homelessness. This study highlights the importance of centering the voices of vulnerable communities to inform future pandemic response for homeless and other underserved and marginalized populations.

## Background

The COVID-19 pandemic has disproportionately impacted vulnerable communities across the country, highlighting existing social inequities further exacerbated by the pandemic. People experiencing homelessness face increased risk and susceptibility to COVID-19 infection and adverse outcomes due to pre-existing comorbidities, barriers to healthcare, socioeconomic precarity, and limited ability to social distance in congregate shelter settings [1, 2]. As a result, heightened risk of transmission and outbreaks in shelters persisted despite decreases in cases among the general population [3]. Shelters and other homeless service providers have taken numerous approaches to mitigate risks, control transmission, and limit outbreaks to minimize adverse outcomes [1, 4, 5].

Previous research on COVID-19 responses from the perspective of homeless service organizations in Indiana found that service providers experienced multilevel challenges during the pandemic, such as limited public health and emergency management guidance and difficulty enforcing safety measures among shelter guests, but also showed innovative responses with systems and staffing in place, along with the support of community and government partners [6]. The COVID-19 response in homeless populations led to improvements in crisis execution and public health protocols such as hand hygiene, social distancing, and quarantine and isolation protocols, and also created initiatives to sustain these programs [6–8]. However, limited adaptable guidance and policies for people experiencing homelessness and service providers have severely strained their response and resources [6]. Others have also discussed COVID-related responses and challenges from the perspective of homeless service providers, including limited availability of testing resources which severely hindered the ability of shelter staff to adequately screen people experiencing homelessness and prevent shelter outbreaks [9]. Furthermore, the economic consequences of the pandemic intensified the strain on low-income populations and evictions disproportionately put those most socially disadvantaged at risk for COVID-19 [10].

While a significant number of studies report trends in coronavirus cases, hospitalizations, and deaths [11], few consider the other numerous impacts of COVID-19 on people experiencing homelessness and scant have explored the impact from the perspective of people experiencing homelessness directly. Among the few studies that have qualitatively explored perspectives of people experiencing homelessness, most have been conducted outside the United States [12–15]. Findings from our previous work in Indiana [6] highlighted the need to hear and learn from people experiencing homelessness directly, in order to holistically understand the impact of the pandemic and to better inform responses that address the specific needs of this uniquely vulnerable population.

Thus, guided by the Socio-Ecological Model [16] which recognizes the interrelatedness of person-environment, this study sought to understand 1) experiences of people experiencing homelessness throughout the COVID-19 pandemic and 2) perspectives of people experiencing homelessness on homeless service organizations’ responses to the pandemic and the impacts of those responses. Awareness of this vulnerable population’s multidirectional needs creates an opportunity to discover motivations, hesitations, and challenges contributing to increased risk, susceptibility, and adverse health outcomes. Understanding these critical factors can better inform future pandemic response as well as interventions to mitigate the long-term impacts of COVID-19 for homeless populations.

## Methods

After exploring local homeless service providers’ and community-based organizations’ responses during COVID-19, we turned to learn from people experiencing homelessness themselves in order to understand how they have personally experienced the pandemic, the challenges they have faced, and the unmet needs that persist. As part of an ongoing community-based participatory research (CBPR) project [6], this study’s recruitment and data collection activities took place at our community partner organization, a transitional housing center in Indiana that serves as the coordinated point of entry for all people experiencing homelessness in the county. The organization includes an engagement center that operates as a day shelter, offering three daily meals, showers, laundry machines, phones, and case management services, and a small night shelter where some but not all guests stay overnight. Using convenience sampling, recruitment involved passive outreach via flyers and general announcements at the shelter. Interested participants were told to contact the phone number on the flyer or to speak to a study team member on site. At no time were people experiencing homelessness approached directly. To be eligible participants had to be age 18 or older, currently experiencing homelessness, and receiving any services from the transitional housing center. There were no pre-determined enrollment targets, as we aimed to capture as many perspectives and narratives from people experiencing homelessness as possible in the six-month study period. Participants received a $25 giftcard to a local grocery store in compensation for their time providing an interview. All study activities took place from January through July, 2021.

An interview guide was developed to understand unique challenges, responses, and experiences faced by people experiencing homelessness during the COVID-19 pandemic. Development of the interview guide was guided by: [1] the Socio-Ecological Model [2]; a review of academic and grey literature conducted to gain insights into COVID-19 responses taken by entities working with people experiencing homelessness, and to identify knowledge gaps that could be informed through interviews; and [3] preliminary findings from our previous research with community-based organizations [6].

Interviews were conducted in-person, in private rooms at the center, by community health workers (CHWs) who live in the surrounding community and serve as health educators in the center, providing health-related education and daily public service announcements during the COVID-19 pandemic. CHWs have relationships, knowledge, and trust with the community they serve and are increasingly being brought into research to better understand the health needs of marginalized populations [17–20]. The CHWs were part of the research team, completed IRB-required trainings on responsible conduct of research and human subjects research, and were further trained on research ethics and data collection by study principal investigators (authors NMR and YR) who have extensive experience conducting CBPR and CHW training interventions. CHWs received extensive training to ensure that people experiencing homelessness understood that participation was voluntary and that involvement, or lack thereof, would not in any way affect their access to center services. Interviews were recorded and transcribed by Otter.ai, a digital scribing platform. Transcriptions were quality checked for accuracy by the research team who also led the analysis. Utilizing a combination of deductive and inductive coding based on the interview guides, two researchers coded each interview independently using Nvivo12, a qualitative coding software, and discussed the interviews as a group to ensure intercoder consistency [21]. Disagreements were brought to the entire research team, including CHWs, and codes and resulting themes were discussed until consensus was reached [22].

Guided by National Institute of Minority Health and Health Disparities (NIMHD) research framework [23], which builds on the Socio-Ecological Model, data was thematically analyzed [24], and themes were organized by levels of influence and domains of influence. Preliminary deidentified findings that highlighted strengths, opportunities, and the challenges of the responses to COVID-19 as perceived by people experiencing homelessness were shared with the study’s community partner as well as other community-agencies that serve this population. Feedback from the community partners helped contextualize and clarify aspects of the findings, and sharing preliminary findings also allowed the community partners to act quickly on identified needs or opportunities to improve service delivery for people experiencing homelessness. This study was approved by the University’s Institutional Review Board (protocol IRB-2020-1488).

## Results

In total 34 people experiencing homelessness (*M*_age_ = 46 years [range 22 to 63]; 65% male; *M* = 3.5 years spent experiencing homelessness [range from 6 months to over 10 years]) participated in semi-structured interviews. Most identified as White (79%) with 6% as Black/African American, 9% American Indian or Alaska Native, and 3% as multiracial. Most (59%) reported having high school or equivalent education and over half (53%) reported no monthly income (6% < than $500, 24% between $500–999, and 18% ≥ $1000). The demographic characteristics of our participant pool are fairly representative of the 2021 point-in-time count results of homeless populations in Indiana [25]. Additional demographic information is presented in Table 1.

**Table 1:** People Experiencing Homelessness: Participant Characteristics

	Participants (*N* = 34)	
	*n*	(%)
Age		
18–39 years	8	24%
40–49 years	11	32%
≥ 50 years	15	44%
Sex		
Male	22	65%
Female	12	35%
Marital status		
Single/never married	14	41%
Divorced/separated	13	38%
Married/partnered	4	12%
Widowed	3	9%
Race		
White	27	79%
Black or African American	2	6%
American Indian or Alaska Native	3	9%
Other or did not respond	2	6%
Ethnicity		
Non-Hispanic	31	91%
Other or did not respond	3	9%
Education		
Some high school or less	9	26%
High school or equivalent	20	59%
Vocational training, college	5	15%
Income per month		
$0	18	53%
< $500	2	6%
$500–999	8	24%
≥ $1000	6	18%
Lifetime years of homelessness		
< 1 year	10	30%
1–3 years	13	38%
4–9 years	8	24%
≥ 10 years	3	9%
Vaccination status / willingness		
vaccinated	7	21%
unvaccinated; willing	8	24%
unvaccinated; unwilling	7	21%
unvaccinated; undecided	11	32%
did not respond	1	3%
Willing to be tested for COVID-19		
yes	27	79%
no	3	9%
undecided	2	6%
did not respond	2	6%
Believe at risk for COVID		
yes	17	50%
no	16	47%
did not respond	1	3%
Experienced COVID-19 (Self-report)		
yes	5	15%
no	16	47%
did not respond	13	38%

**Fig. 1:**
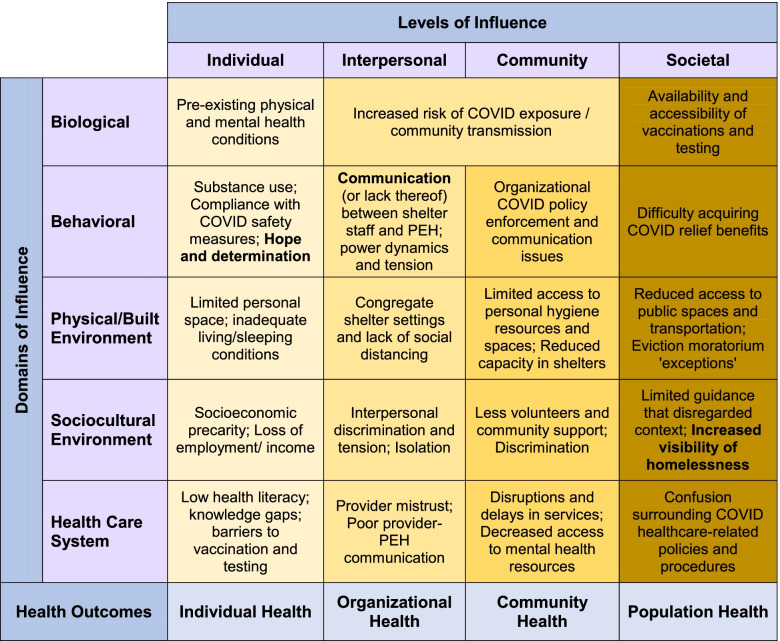
Positive (**bold**) and negative factors affecting COVID-19 risks and outcomes among people experiencing homelessness (PEH). Based on the NIMHD Health Disparities Research Framework [23]

Qualitative content analysis and resulting themes were organized by level of influence: individual, interpersonal, community, and societal (Fig. 1).

### At the individual level, across all domains of influence, people experiencing homelessness face unique challenges that heightened their vulnerability during the COVID-19 pandemic

Participants spoke of concerns about their biological risk of severe COVID-19 due to their pre-existing health conditions. One participant shared: *“I know it, if I get it I’m dead, because the way my lungs and my health and stuff are—it’s killing athletes, why wouldn’t it kill me? I don’t eat right, I smoke cigarettes, I’ve been a drug addict my whole life. If it’s killing athletes, it’s definitely going to kill me” (Participant 009).* Others shared how the pandemic amplified existing mental health disorders: *“Just being depressed… there’s nothing to do, and people aren’t meeting and this just like sucks” (Participant 028).* Others shared fears that their behaviors such as substance use would increase their COVID susceptibility and their risk for adverse COVID-19 outcomes. One noted an increase in smoking, *“I did pick up cigarettes again since I’ve been here [shelter], and I had stopped for six months, and now I’m smoking again about a half a pack a day… “ (Participant 018).* While others shared how the pandemic led some to battle with their substance abuse, *“the urge to want to use went up” (Participant 028).*

By contrast, some participants expressed hope that the pandemic could offer them a chance to start over and because the pandemic forced many people to lose their jobs it could create opportunities for them to find work, “*In a sense, COVID may have actually helped some of us homeless, because it kind of ground to a halt, something that is that we’ve been missing when it’s been flying by us, we haven’t been able to put the pieces together and actually get on and get in the grind. And so in that sense it’s helped because it’s, it’s helped is to put a lot of people in the industry as well. So in that respect, it can help because everybody has to start over and look at that job again. I had, I’m starting over*” *(Participant 022)*. One participant described seeing themself as a survivor with determination of overcoming challenges, *“My mental attitude hasn’t changed... even when I had COVID, I wasn’t like sad or angry or anything… I know I’m going to get through this, I know I’m going to survive” (Participant 017).*

Reflecting on the physical/built environment, specifically the impracticality of social distancing in the shelter a participant shared: “*I don’t think the six feet social distancing applies here in the homeless community, because people are within two feet when we eat 3 times a day, or when they go outside and smoke cigarettes, they’re within four feet, or when they sleep inside the shelter, they’re within four feet*” *(Participant 018)*. Indeed some felt that the shelter’s congregate living conditions increased their risk of infection, *“this [shelter] would be the best place to catch COVID” (Participant 018).* As many lost employment due to COVID-19, this increased socioeconomic precarity which forced many into homelessness. *“It affected our household heavily, we lost a lot of money because of it, and not having that income, having employment shutdown forced her to lose her job, and then find another job, only to have somebody in her department that she was working at test positive for COVID, and then have everybody from that entire department quarantined for two weeks which stopped more income. It’s ultimately led to a really negative downward spiral… Even emergency savings that we had set up to prevent any sort of homelessness in the first place. I don’t think that the relief packages that they’ve sent out have offset the sheer cost to the lower class or working class American to stop most of these financial emergencies from happening” (Participant 018).* Furthermore, participants noted how increasingly difficult it was to find employment during the pandemic, *“with this stuff going around, it’s hard. People don’t want to hire nobody” (Participant 003).*

Evidence of low COVID-19 health literacy and knowledge gaps emerged with only 50% of all participants believing they were at risk for COVID-19 (Table 1) and through participant narratives, some sharing, *“I don’t know anything about COVID…” (Participants 020)* and *“I was in prison, so I don’t know too much” (Participant 015).* This lack of knowledge affected COVID risk perceptions among people experiencing homelessness with some expressing fear: *“I’m scared to death, about getting this COVID, it really, really makes me paranoid” (Participant 009)*, some feeling hopelessness, *“… even if I did get it, I wouldn’t care. I mean…my life, besides, the quality of it is not good, so…it doesn’t matter*” *(Participant 005)*, and others sharing feelings of indifference and disbelief: “*I don’t know anyone who has died from it. My girlfriend doesn’t know anyone who has died from it. So, I don’t trust the media…I think that maybe this whole thing was blown out of proportion. But I know that it made my homeless crisis that much more difficult” (Participant 023).*

Poor COVID knowledge also affected willingness by people experiencing homelessness to adopt COVID prevention measures like vaccination and testing. At the time of the study, most participants (79%) shared that they were willing to be tested for COVID-19, however only 21% of participants were vaccinated, 24% were unvaccinated but expressed a willingness to be vaccinated, 21% were unvaccinated and unwilling, and 32% were undecided (Table 1). Faith followed by fear were among the most common reasons for their unwillingness to be vaccinated, as one participant noted, *“I’m dead set in not getting it cause I trust in God. I mean, if, if I want to say I trust in God and then turn around and.. wouldn’t that be taking my faith away? Wouldn’t that be destroying my own faith?” (Participant 029).* Some shared being fearful that the vaccine would make them sick, *“I’ve had, like, the flu vaccine and I…I don’t like it because it’ll actually make you sick. It’s the actual flu itself that they’re injecting you with… I don’t want to take no shot” (Participant 005),* and others feared that the swabbing procedure would be painful, “*I will not take a giant Q-tip up on my nose. Everybody else says it’s painful and very uncomfortable” (Participant 024).* Moreover, some shared being unwilling because they feared being quarantined if they tested positive.

### At the interpersonal level, poor communication and discrimination led to misunderstandings and tension between people experiencing homelessness and homelessness service providers

Some participants acknowledged that the pandemic made the work of shelter staff, given all the uncertainty and limited resources, quite difficult: “*they’re [staff] doing the best they can with what they have*” *(Participant 004)*. Others expressed appreciation towards staff who enforced mask wearing, *“Every time they turn around, they’re telling somebody ‘put your mask on, if you’re not eating put your mask on’…Yeah, they take it very serious, it’s a good thing” (Participant 034).* In fact, one newly homeless individual described how quickly staff alerted him of the already in place COVID-19 policies of mask wearing, sanitizing, and social distancing: “*I was pretty much baptized right into what was already going on...Wearing masks. Social distancing. The hand sanitizers*” *(Participant 020)*.

In contrast, many participants described how authoritative power dynamics and poor staff-to-client communication led to misunderstandings and tension between people experiencing homelessness and service providers that often resulted in poor compliance of COVID safety policies. One participant shared feeling frustrated by staff who instead of explaining the rationale behind mask-wearing simply threaten to remove people experiencing homelessness from shelter premises for non-compliance: *“If we don’t have this [mask] on, they say ‘get your mask up or you’re kicked out,’ Now see that’s wrong. To me, that’s wrong. They need to be more specific and explain why we need to be wearing a mask rather than just saying ‘put your mask up’ and [if you] don’t…you get kicked out. Where you going? To sleep outside. It’s cold and if you don’t have adequate clothing, you’re screwed...I see a lot of injustice in this system now*” *(Participant 005)*. Interestingly, when asked about their sources for COVID-19 information, most participants noted learning from social media, *“Mostly through Facebook… I don’t watch the news. Obviously we don’t have TVs here, (Participant 005)”* from other people experiencing homelessness, *“Word of mouth. A homeless guys word is bond and I met some of the most loyal people in this world on the street” (Participant 022),* various news outlets, and CDC YouTube updates, however none of the participants indicated garnering COVID-19 information from shelter staff.

Adding to the tension between people experiencing homelessness and service providers was a sense that staff often did not follow the rules themselves, “*I don’t think they know how to handle this virus situation, you know? When we go to lunch, they say ‘six feet apart’. You’re not six feet apart behind that thing [plexiglass] back there. You know, that’s what we’re seeing. You know you guys aren’t no better than us…I mean instead of yelling at us homeless people. You know, we know what’s going on. We know the risk. We’re around each other every day, you know... Quit saying “stay six feet apart,” you know. I mean you’re not six feet apart behind that cubicle, you know. I mean it’s like they’re contradicting themselves*” *(Participant 006)*. Moreover, this participant felt that the staff’s non-compliance was putting them at risk for COVID, *“…how are they [the staff] not spreading the virus? You know what I’m saying? …you guys supposedly better than us? And you guys are dealing with the same thing [pandemic] every day, other than being homeless…We know to stay six feet apart, you know, but it doesn’t matter. We’re here every day with each other, interact every day. You know, save your breath, quit bitching… I mean if that’s the case, they’re not doing anything themselves to help… if we’re supposed to be ten…six feet apart, so should they…who’s going to yell and scream at them?” (Participant 006).* Others shared that the tension between staff and people experiencing homelessness was affecting their mental health “*Just probably my aggravation levels have gone up, because I just feel like somebody is trying to control me and tell me what to do, what I can’t do” (Participant 020).*

In addition to concerns related to how staff handled COVID safety practices, participants expressed skepticism and concern towards the ways staff handled shelter closures when clients tested positive for COVID-19, *“… they put the building on lockdown stopping new people from coming in... And I noticed that the health department was quick to lock down the buildings, but they weren’t quick to lift the lock down with a false positive happening and being reported, which makes me question both the response and the direction that they’re taking with the lockdown. As I’m already noticing people coming in looking for services and being turned away as a result of a false positive*” *(Participant 018)*. Similarly, another shared, *“what upsets me is like for instance, if you’ve not been here since [start of lockdown]… you will not receive services. So, what are you supposed to do? You’re out on the street” (Participant 005).* One participant went on to share how a lockdown further exacerbated his homelessness status, *“when the building was closed down and [they were] not accepting new clients because of COVID, it forced us to use the last bit of our savings on a hotel… putting us in a really, really bad financial spot” (Participant 018).*

Tensions between people experiencing homelessness themselves were also reported. The congregate shelter setting and limited personal space led to tension between some shelter guests, exacerbated by interpersonal discrimination where some participants described other people experiencing homelessness as *“a stubborn bunch” (Participant 020),* stating that *“some people just do not care at all about other people and they just cough right in their face and wipe their snot everywhere… sometimes you have people basically touching you, or touching your backpack or whatever clothes. Maybe it’s an accident maybe they’re doing it on purpose, who knows” (Participant 015).*

### At the community level, closures and service disruptions restricted access to usual spaces, routines, and ability to meet basic needs

All participants spoke in detail about how COVID-related closures in the community had affected their ability to meet basic needs on a daily basis. Some had great difficulty finding spaces to shelter or even just to be. *“Because of COVID there’s a lot of smaller restaurants that people would be able to go to get coffee, utilize WiFi, and have a place to stay warm. So you either have to stay at [shelter] for several hours, and do nothing. Or find somewhere warm to wait until the shelter opens up. Covid has kind of messed a lot of things up” (Participant 018).* Participants discussed difficulty finding spaces or resources for personal hygiene. *“I couldn’t find a bathroom anywhere. There was no access. No public access bathrooms anywhere. I couldn’t shower, bathe, take care of myself because they shut all the water down in the public parks. I mean, it’s just, it’s been difficult in that regard” (Participant 023).* Others described challenges they faced in finding places to relax or destress away from the shelter*. “When the pandemic hit, it’s like everything shut down. So, it was very hard for me to find stuff, find places to go where if my senses are overstimulated, it is very hard for me to find a place to where I, you know, I could get myself de-stressed” (Participant 030).* Another shared, *“Well, a lot of times, when I go to the library, I like to spend a lot of time in the library and it helps me to… because if I’m, if my autism is really flaring and I’m overstimulated, going to the library helps me to kind of de-stimulate basically... [now] they only let you be in there for 30 minutes” (Participant 011).* One individual explained how the closures caused changes in their routine, *“Especially during the pandemic, it’s been, it’s been a bit of a challenge for me. You know I’m used to being able to go you know here, there, everywhere, you know, and such, you know, without any issue. But the pandemic is, you know, kind of caused us, maybe a little hamper and whatnot you know, but I’m learning how to adapt around it” (Participant 030).* Others shared that COVID was one more challenge on top of everything else they faced on a daily basis. For example, a formerly-incarcerated participant shared, *“I caught COVID. When I was laying on the floor in the shelter after trying to do everything right to get parole, which I did…” (Participant 022).*

Regarding shelter-specific organizational responses to the pandemic, some participants expressed a positive reaction to the accessibility of sanitizing products along with more frequent bathroom cleaning in the shelter, stating, “*I feel like the hand sanitizer everywhere. That’s awesome. I think that helps*” *(Participant 028).* Another stating, “*I like how [the shelter] nightly have cleaners to go clean the bathroom, like it’s never been cleaned before*...” *(Participant 034)*. Others commented on the lack of consistent resource availability available in restrooms such as soap, toilet paper, and paper towels, *“…the people taking all the paper towels or soap dispensers being empty…generally you can’t get in the bathroom in here anyway, so I just mostly use the hand sanitizer... they don’t always have toilet paper, they don’t always have paper towels. It seems like the soap dispensers aren’t being filled*” *(Participant 020)*. The increase in shelter demands because of the pandemic further strained the already limited resources leading to longer wait times for restrooms, one participant stating, “*It’s so bad here in the morning with these bathrooms that I got to take the number seven out to Walmart and use the bathroom out there. I don’t even bother trying to come here*” *(Participant 023)*.

Two participants felt that during the pandemic, relief resources allowed for continued or even increased accessibility to services. One stating that services *“Became easier to get. Felt it.” (Participant 008),* and another sharing, *“I actually got housed within like a couple weeks” (Participant 024).* However, the majority of participants described how COVID-related service disruptions severely restricted or delayed access to key services. *“I had to continue to live on the streets, even though it was four-degree weather out because of the fact that the [shelter], had a case of COVID....” (Participant 022).* One participant shared, *“[behavioral health providers] used to come, but they don’t no more because of the COVID thing” (Participant 012).* One participant described the delays he experienced in accessing necessary paperwork, “[*I was] referred to [homeless service organization] to get my green card and [organization] was shut down… somebody COVID in there so nothing happened until January… that I finally was able to get in there into the zoom thing, meeting with them got, you know the application kind of filled out and everything…” (Participant 017)*.

In addition to service disruptions, participants also described the notable decrease in community support from volunteers, *“And with the virus and all, what it did, it brought the families closer together at their homes. It makes some of the [people] or the churches or some that used to help nonexistent. They just don’t want to take the time to do it or take the time to help” (Participant 027).* In contrast, some participants spoke of increased visibility of homelessness as a silver linings for homeless communities resulting from the pandemic, *“How is [covid] affecting me? it’s affecting everyone in the whole country but even more so is the homelessness. I think COVID has actually helped empower some homeless communities because of the fact that some of the commercials are put out by the big conglomerate businesses that are really enlightening and really heartfelt and very spot on” (Participant 022).*

### At a policy level, people experiencing homelessness continue to be neglected in ways that made pandemic relief resources largely inaccessible

Participants described how unclear guidance on COVID policies and stimulus funding led to overwhelming confusion and inability to access relief resources. *“I didn’t get my last year’s tax returns, or my stimulus check, so I have to go to the IRS… Because you can’t call them nobody answers the phone there either… The system has become so inadequate [for] people like us, the homeless. They don’t care. The government does not care…the ones that are getting income, the ones that are on unemployment, they’re getting the stimulus checks. But true people that are homeless ain’t getting shit. No address…well I mean they can use this [shelter’s] address, because this [is] where we have our mail sent. But I don’t know. It’s just…overwhelming” (Participant 005).* Those who lost jobs expressed difficulty accessing unemployment benefits*. “...Even to hop online and do my unemployment stuff is just, it was just so difficult for me” (Participant 017).* Individuals also noted a lack of understanding regarding unemployment resources. *“I believe that’s where the Cares Act comes in. A lot of people don’t understand the care’s act. It’s unemployment insurance that is not just for people that were employed at that time, but it was people that for myself, they couldn’t get employment because everything shut down....” (Participant 022).* Participants also expressed confusion surrounding COVID-19 policies and procedures related to healthcare and insurance, *“I didn’t even know it was still valid [health insurance] or I would have went to the frickin hospital. So I didn’t get any kind of testing I didn’t get any kind of treatment” (Participant 017).* Furthermore, despite the federal moratorium on evictions, this participant shared how they became homeless during the pandemic, *“…Even though was a moratorium on evictions for nonpayment, [landlord] evicted me for having somebody else living there which is against the lease. So, I became homeless again” (Participant 017).*

Local COVID-related policies, including “stay-at-home” orders, mask mandates that meant people experiencing homelessness were never able to be without a mask indoors, and transit rules were not clearly communicated to people experiencing homelessness and often disregarded their specific needs and context. One participant shared, *“We were riding the buses for free because they didn’t want to handle the change… Alright. Nothing has changed. The virus is still there. So it’s like, why we ain’t riding the buses for free now?...Yeah, you know and they’re making us pay now, and it’s like, the virus hasn’t changed” (Participant 006).*

## Discussion

This study explored the impacts of the COVID-19 pandemic on homeless populations in the US through first-hand accounts from people experiencing homelessness in Indiana. Across all domains of influence (biological, behavioral, physical environment, sociocultural environment, health care system), interviews with PEH revealed multilevel factors affecting their susceptibility to COVID-19 and other adverse outcomes of the pandemic. While existing research has surveyed people experiencing homelessness to understand specific COVID-related issues such as loneliness and isolation [13, 14], mental health and substance use [26], and attitudes towards vaccinations and testing [8, 27, 28], there have been limited efforts to provide accounts from people experiencing homelessness themselves, in a way that centers the voices of the most affected to understand direct impacts of the pandemic on this vulnerable population. This community-based, qualitative study explored narratives of lived experiences and perspectives about being homeless during the pandemic. To date, several studies have garnered perspectives from homelessness service providers and reported on numerous challenges these frontline workers faced throughout the pandemic as well as the complex and innovative ways they navigated and responded to these challenges [6, 29, 30]. In many aspects, narratives of people experiencing homelessness supported provider accounts, particularly around this population’s pre-existing physical, mental, and behavioral health conditions that were exacerbated by COVID-related service disruptions and multilevel challenges that made safety measures like social distancing difficult and often impossible.

The absence of the voices of people experiencing homelessness in research can miss a more direct and nuanced understanding of motivations, knowledge, attitudes, and beliefs that contribute to challenges. Indeed, our findings highlighted several important and mitigable issues that had not come up in our previous work which focused solely on provider perspectives. For instance, many participants spoke of communication issues between shelter staff and guests that led to poor understanding and low compliance of COVID-related safety measures. Specifically, participants felt that little to no effort had gone into informing or educating them about COVID-19 and they were rarely offered rationale for new shelter rules such as mask-wearing and social distancing. Moreover, they shared that staff neither explained nor modeled expected behavior, but instead “contradicting themselves”, communicated by “yelling”, “trying to control”, or “threatening to kick out” shelter guests. Interviews with people experiencing homelessness also revealed important knowledge gaps and misinformation surrounding COVID-19 that were made worse by a lack of reliable information sources. Not a single participant mentioned shelter staff as a source of COVID-related information, instead indicating that sources were often word-of-mouth and social media. Furthermore, interviews emphasized key policy failures that made state and federal pandemic responses especially neglectful of and even harmful to homeless communities including mask mandates, stay-at-home orders, and closures of public spaces and transportation, which disregarded the context and unique needs of people experiencing homelessness. Other policies such as eviction moratoriums contained loopholes and exceptions that failed to protect this vulnerable population. Lack of tailored guidance also led to confusion among people experiencing homelessness surrounding COVID healthcare-related policies and procedures and created substantial barriers to acquiring relief resources and stimulus benefits.

This study had several limitations. First, as the interview participants were recruited only from one county in Indiana, we cannot assume that our results are representative of the larger population of people experiencing homelessness in other areas of the US. Because recruitment and eligibility was restricted to guests receiving services at a homelessness service organization, it is possible that this study is missing the perspective of unsheltered people who may not be accessing any support services and thus remain particularly vulnerable. In addition, we relied on convenience sampling for this study, which depends on the motivation of those who participate in the research and thus can introduce motivation bias. Nonetheless, our findings reveal numerous opportunities for multilevel interventions and improved disaster response for homeless populations that may be useful for other contexts. At the individual level, this work highlights the imperative for outreach, education, and navigation of PEH through healthcare and social welfare systems. Community health workers and other types of outreach workers have served as essential links between underserved populations and health and social services both during and long before the pandemic [18, 19, 31, 32]. Hiring and deploying trusted individuals with lived experience or knowledge of the community could be the key to pandemic response in homeless populations by providing education, testing, access to vaccines, and navigation of relief programs, stimulus checks, etc. At the organizational level, training interventions for shelter staff and other homelessness service providers on implicit bias, cultural competency, effective communication, conflict resolution, mental health and substance abuse, and COVID-19 mitigation in shelters [33–37], could allow for better communication skills, strategies, and improved ability to meet the needs of people experiencing homelessness. At the societal and policy level, federal and state guidance and policy must be inclusive of our most vulnerable populations and tailored to their local contexts, which can only be achieved through meaningful engagement of members of these vulnerable communities. People experiencing homelessness must be engaged, listened to, and counted in a meaningful and participatory way. The majority of federally reported COVID-19 outcomes in homeless populations focused on numbers of cases and deaths, and disregarded both the complexities that made those counts inaccurate, as well as the enormous range of other impacts these communities faced [38]. People experiencing homelessness must also be protected from policy loopholes and other exceptions that exacerbate inequities and perpetuate a vicious cycle of falling through cracks. Stronger eviction prevention measures and policies to prevent homelessness and provide affordable housing, including permanent supportive housing, are increasingly critical beyond the COVID-19 pandemic [39–41].

Despite the overwhelming challenges faced by homeless populations, participants’ also described numerous elements that helped them cope, overcome, and even grow despite the traumas and significant stressors, with some indicating being hopeful that the pandemic might offer them an opportunity for a fresh start. There is increasing evidence that demonstrates that supportive programs can assist people to exit homelessness [42–44], yet without centering these efforts on the voices of those most affected these efforts will continue to fall short. Further research is needed to enable the U.S. to create a system that is person-centered. These efforts must provide not only a better understanding of the unique and multidirectional needs of people experiencing homelessness, but also move beyond a deficit model towards one that identifies supportive protective factors so programs and policies can not only help individuals exit homelessness but also strive to reduce risk of homelessness.

## Data Availability

The datasets of the current study are available from the corresponding author on reasonable request.

## Abbreviations

CARES: Coronavirus Aid, Relief, and Economic Security
CBPR: Community-based participatory research
CHW: Community Health Worker
NIMHD: National Institute of Minority Health and Health Disparities
PEH: People experiencing homelessness
